# Cuproptosis-related gene identification and immune infiltration analysis in systemic lupus erythematosus

**DOI:** 10.3389/fimmu.2023.1157196

**Published:** 2023-05-29

**Authors:** Wuquan Li, Xiaoran Guan, Yong Wang, Yan Lv, Yuyong Wu, Min Yu, Yeying Sun

**Affiliations:** ^1^ College of Pharmacy, Binzhou Medical University, Yantai, China; ^2^ College of Life Science, Yantai University, Yantai, China

**Keywords:** systemic lupus erythematosus, WGCNA, machine learning, immune infiltration, biomarker

## Abstract

**Background:**

Systemic lupus erythematosus (SLE) is an autoimmune disease characterized by loss of tolerance to self-antigen, autoantibody production, and abnormal immune response. Cuproptosis is a recently reported cell death form correlated with the initiation and development of multiple diseases. This study intended to probe cuproptosis-related molecular clusters in SLE and constructed a predictive model.

**Methods:**

We analyzed the expression profile and immune features of cuproptosis-related genes (CRGs) in SLE based on GSE61635 and GSE50772 datasets and identified core module genes associated with SLE occurrence using the weighted correlation network analysis (WGCNA). We selected the optimal machine-learning model by comparing the random forest (RF) model, support vector machine (SVM) model, generalized linear model (GLM), and the extreme gradient boosting (XGB) model. The predictive performance of the model was validated by nomogram, calibration curve, decision curve analysis (DCA), and external dataset GSE72326. Subsequently, a CeRNA network based on 5 core diagnostic markers was established. Drugs targeting core diagnostic markers were acquired using the CTD database, and Autodock vina software was employed to perform molecular docking.

**Results:**

Blue module genes identified using WGCNA were highly related to SLE initiation. Among the four machine-learning models, the SVM model presented the best discriminative performance with relatively low residual and root-mean-square error (RMSE) and high area under the curve (AUC = 0.998). An SVM model was constructed based on 5 genes and performed favorably in the GSE72326 dataset for validation (AUC = 0.943). The nomogram, calibration curve, and DCA validated the predictive accuracy of the model for SLE as well. The CeRNA regulatory network includes 166 nodes (5 core diagnostic markers, 61 miRNAs, and 100 lncRNAs) and 175 lines. Drug detection showed that D00156 (Benzo (a) pyrene), D016604 (Aflatoxin B1), D014212 (Tretinoin), and D009532 (Nickel) could simultaneously act on the 5 core diagnostic markers.

**Conclusion:**

We revealed the correlation between CRGs and immune cell infiltration in SLE patients. The SVM model using 5 genes was selected as the optimal machine learning model to accurately evaluate SLE patients. A CeRNA network based on 5 core diagnostic markers was constructed. Drugs targeting core diagnostic markers were retrieved with molecular docking performed.

## 1 Introduction

Systemic lupus erythematosus (SLE) is an autoimmune disease (1) (AID) featuring loss of tolerance to self-antigen, autoantibody production, and abnormal immune response. It affects multiple organs, such as skin, joints, kidneys, lungs, and hearts (2), severely interrupting work, normal routine, and physical and mental health. The pathogenesis of SLE is not clear. Genetic and environmental factors and viral infection are considered possible pathogenic factors (3–5). So far, specific drugs for SLE are very scarce, and SLE patients are still treated with traditional anti-inflammatory drugs, immunoregulatory drugs, and corticosteroids, often accompanied by adverse events (AE). Therefore, investigating molecular characteristics and mechanisms of SLE has substantial implications for providing new strategies for SLE diagnosis and treatment.

Copper serves as a cofactor in many enzymes and has important physiological functions in vital activities (6). Normal cells have a quite low copper concentration, and they prevent the accumulation of free intracellular copper ions mainly by active transport mechanisms, thereby sustaining copper homeostasis (7, 8). Copper imbalance leads to oxidative stress (9), aberrant autophagy (10), etc., thereby inducing various copper/copper ion-associated diseases. Cuproptosis is a copper-dependent programmed cell death form, and its mechanism is different from apoptosis, pyroptosis, necrosis, and autophagy. In cuproptosis, copper directly binds with lipoylated proteins in the tricarboxylic acid (TCA) cycle, leading to lipoylated protein aggregation and following iron-sulfur cluster loss, inducing proteotoxic stress and eventually cell death (11). Research showed that ferroptosis in neutrophils leads to the occurrence of SLE, and the mechanism is by promoting cAMP response element modulator CREM binding with glutathione peroxidase 4 (GPX4) promoter to downregulate GPX4 expression (12). The significance of copper homeostasis in immune infiltration has been reported in relevant studies recently. It was reported that copper chelation in macrophages can eliminate lysyl oxidase-like 4-mediated programmed death-ligand 1 presentation, thereby suppressing cell immune escape (13). However, currently, cuproptosis’s role in the initiation and development of SLE is still not clear. Hence, elucidating cuproptosis’s role in SLE has considerable implications.

Machine learning is being widely applied in the medical field, particularly in disease diagnosis, prediction, and treatment. With its high efficiency in thousands of types of diseases, machine learning can be divided into three main types: supervised learning (14), semi-supervised learning (15), and unsupervised learning (16) each being used to train artificial intelligence models with different types of data in different circumstances. Among these, the prediction of disease biomarkers is one important application of machine learning in disease prediction, which can provide doctors with more accurate diagnostics and treatment decisions, ultimately increasing the cure rate and prognosis of the disease. The future of medicine will place increasing emphasis on machine learning. For instance, it could be used to quickly and accurately detect lung cancer in chest CT scans, improving patient survival rates (17). Machine learning can also predict the risk of heart disease in electrocardiograms (18), detect early retinal lesions using semi-supervised learning techniques (19), and assist in diagnosing Alzheimer’s disease (20). By incorporating machine learning, we can gain better insight into the underlying mechanisms of diseases and provide potential targets for future treatments. However, machine learning is just a tool, and we still need to combine it with practical disease research to effectively address specific problems.

In this study, we analyzed differentially expressed cuproptosis-related genes (CRGs) between healthy people and SLE patients using the Gene Expression Omnibus (GEO) database and conducted a bioinformatics analysis of immune characteristics. Based on the CRG expression profile, we assigned SLE patients to two cuproptosis-related clusters and further compared the CRGs of the two clusters. Subsequently, we identified key modules associated with SLE initiation using the weighted gene co-expression network analysis (WGCNA) algorithm. Furthermore, we constructed a predictive model that can reveal the prognoses of patients with different molecular clusters by comparing multiple machine-learning methods. Nomogram, calibration curve, decision curve analysis (DCA), and an external dataset were adopted to verify the performance of the predictive model. Additionally, we established a competing endogenous RNA (CeRNA) regulation network, and selected drugs that act on key biomarkers using the CTD database, which were used as candidate drugs for SLE.

## 2 Materials and methods

### 2.1 Data acquisition and preprocessing

**Figure 1:**
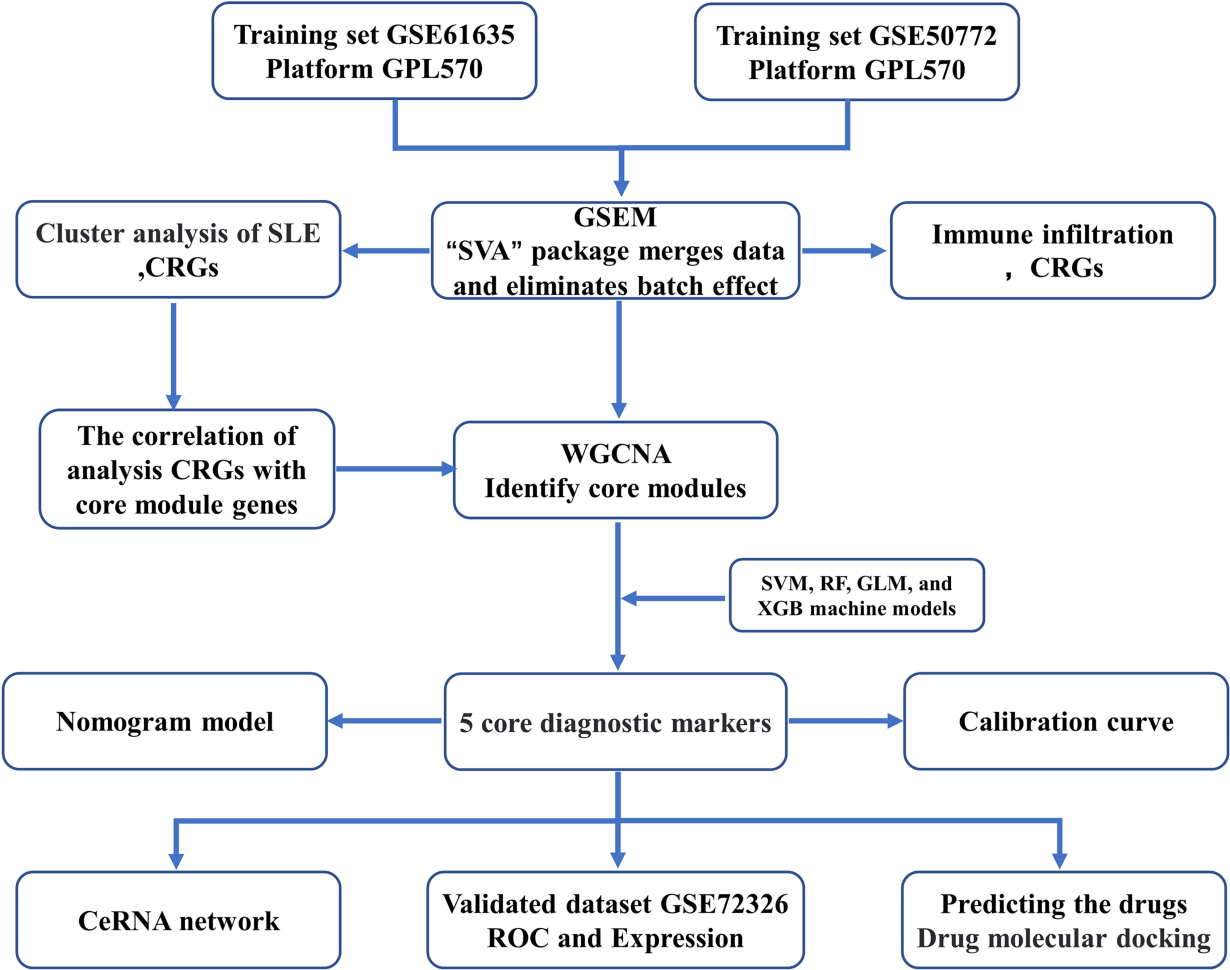
The Study flow charts.

The flow chart of this study is shown in **Figure 1**. The GSE61635 (21), GSE50772 (22), and GSE72326 (23) datasets were retrieved from the GEO database (**Table 1**), and CRGs (11) were collected from published studies. GSE61635 and GSE50772 were merged as one dataset (GSEM) serving as the training cohort since the two datasets were obtained from the same platform, and the GSE72326 dataset was used as the validation cohort. The raw data were normalized and annotated with background subtracted, and batch effects from the merged dataset were removed using the “SVA” package.

**Table 1:** Basic Information of Gene Expression Profiling.

GEO ID	Platform	Samples	Number of Controls	Number of Cases	Country	Year	Author
Training set							
**GSE61635**	GPL570	110	30	80	USA	2015	Greidinger EL
**GSE50772**	GPL570	81	20	61	USA	2015	Kennedy WP
Validation set							
**GSE72326**	GPL10558	177	20	157	USA	2022	Chiche L
**CRGs**	NFE2L2, NLRP3, ATP7B, SLC31A1, FDX1, LIAS, LIPT1, DLD, DLAT, PDHA1, PDHB, MTF1, GLS, CDKN2A, DBT, DLST						Tsvetkov

### 2.2 WGCNA

WGCNA was performed using the R “WGCN”package (24). The optimal value of the weighting parameter in the adjacent function was obtained using the pickSoftThreshold function and served as soft-thresholding power for following network construction (25). Subsequently, weighted adjacency matrices were established, and gene modules were created by hierarchical clustering based on a 1-TOM dissimilarity matrix (26). Each module was assigned a unique color identifier, and the module eigengene represents the expression profile of the entire module. Module–disease state relationships represent module significance (MS), and gene significance describes a gene’s correlation with a phenotype.

### 2.3 Predictive model construction based on multiple machine-learning methods

Machine-learning predictive models include the support vector machine (SVM) model, random forest (RF) model, generalized linear model (GLM), and extreme gradient boosting (XGB) model. The SVM algorithm seeks the separating hyperplane that yields the maximal margin to discriminate positive instances from negative instances (27). The RF is an ensemble learning method yielding several independent decision trees to predict classification or regression (28). The GLM is an extension of the multiple linear regression model, and it can flexibly assess the relationship between normally-distributed dependent characteristics and continuous or categorical independent characteristics (29). XGB is a collection of gradient-boosted trees that can carefully compare and analyze complexity and classification error (30). The four machine learning models were explained using the “DALEX” package, and residual distribution and feature importance among the models were visualized. The AUC of the ROC curve was visualized using the “pROC” R package. Eventually, we confirmed the optimal machine learning model and selected the top 5 factors as SLE-related key predictors.

### 2.4 Nomogram construction and validation

A nomogram was established to evaluate SLE occurrence in clusters using the “RMS” R package. Each predictor contributes to a score, and the “total score” represents the sum of the score of the above predictors. The calibration curve and DCA were adopted to evaluate the predictive performance of the nomogram.

### 2.5 Immune cell infiltration analysis

The CIBERSORT algorithm (https://cibersort.stanford.edu/) was performed to estimate the relative abundance of 22 kinds of immune cells for each sample using the LM22 signature matrix and gene expression data. CIBERSORT uses Monte Carlo sampling to obtain a p‐value for the deconvolution of each sample. Only samples with P < 0.05 were considered to have accurate immune cell fractions, and the sum of the 22 immune cell compositions in each sample was 1 (31).

### 2.6 lncRNA-miRNA-mRNA CeRNA network construction

miRNA-miRNA interactions were predicted using TargetScan (http://www.targetscan.org), miRDB (http://www.mirdb.org/), and miRanda (http://www.microrna.org/) databases, and miRNA-lncRNAs were predicted using the SpongeScan database (http://spongescan.rc.ufl.edu/). Based on lncRNA-miRNA-mRNA interactions, a ceRNA network was constructed using Cytoscape software (3.8.2).

### 2.7 Identification of candidate small-molecule drugs

Drugs corresponding to those diagnostic biomarkers were retrieved using the CTD database (http://ctdbase.org/) to confirm potential SLE drugs. Drug-gene network was constructed and visualized. Molecular docking was performed between selected drugs and sites of key biomarkers using Autodock vina software V1.1.2, and the results were visualized using Pymol V3.9.2.

## 3 Results

### 3.1 CRG expression and immune infiltration analysis in SLE patients

**Figure 2:**
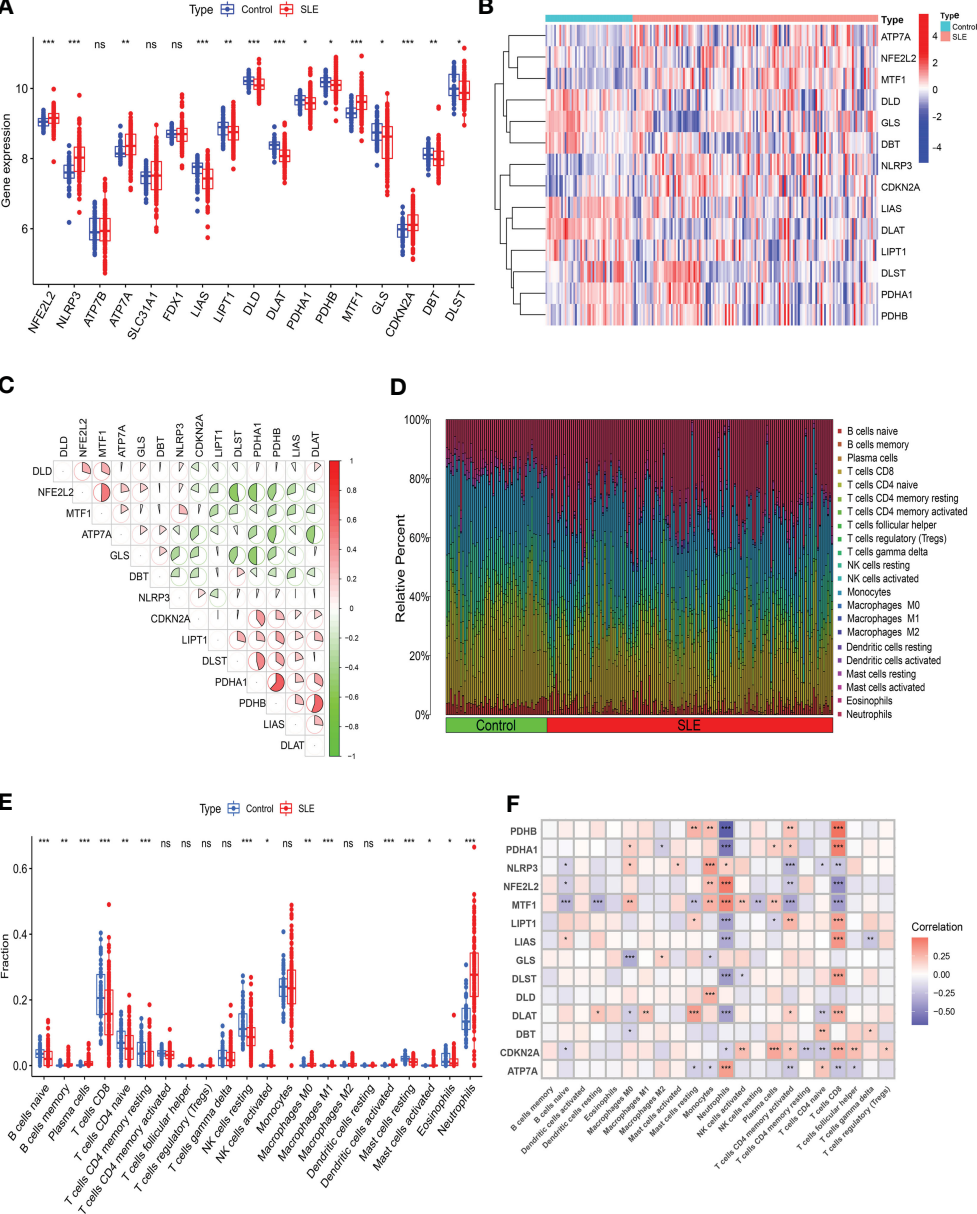
Identification of the expression of CRGs and Immune infiltration analysis in SLE. **(A)** Boxplots showed the expression of 14 CRGs between SLE and Control. **(B)** The expression patterns of 14 CRGs were presented in the heatmap. **(C)** The correlation of CRGs. **(D)** The relative abundance of immune cells between SLE and Control. **(E)** Boxplots showed the difference of Immune infiltration in SLE and Control. **(F)** The correlation analysis between 14 CRGs and immune cells. (* *p*≤ 0.05, ** *p*≤ 0.01, *** *p*≤ 0.001, ns, no significance).

We evaluated the expression profile of 17 CRGs in SLE and normal Control samples using the merged dataset GSEM to investigate CRGs’ biological functions in SLE patients. SLE samples presented higher expression of NFE2L2, NLRP3, ATP7A, MTF1, and CDKN2A genes and lower expression of LIAS, LIPT1, DLD, DLAT, PDHA1, PDHB, GLS, DBT, and DLST genes versus the Control group (**Figures 2A, B**). Subsequently, we conducted correlation analysis for differentially expressed CRGs (**Figure 2C**) to probe CRGs’ role in SLE development. Notably, some CRGs like PDHB and PDHA1 exhibited synergistic effects (R = 0.62). Meanwhile, NFE2L2 and DLST displayed significant antagonistic effects (R = 0.53). Moreover, we performed an immune infiltration analysis to illustrate the difference in immune systems between the normal Control group and SLE patients. The CIBERSORT algorithm revealed a significant distinction in the proportions of 22 kinds of immune cells between the Control and SLE groups such as neutrophils, plasma cells, CD8^+^ T cells, naive CD8^+^ lymphocytes, M1 macrophages, activated dendritic cells (DC), resting mast cells, etc. (**Figures 2D, E**), suggesting that immune system alteration might be the primary cause of SLE occurrence. Additionally, correlation analysis demonstrated that neutrophils and CD8^+^ T cells were correlated with cuproptosis (**Figure 2F**).

### 3.2 Identification of SLE cuproptosis cluster

**Figure 3:**
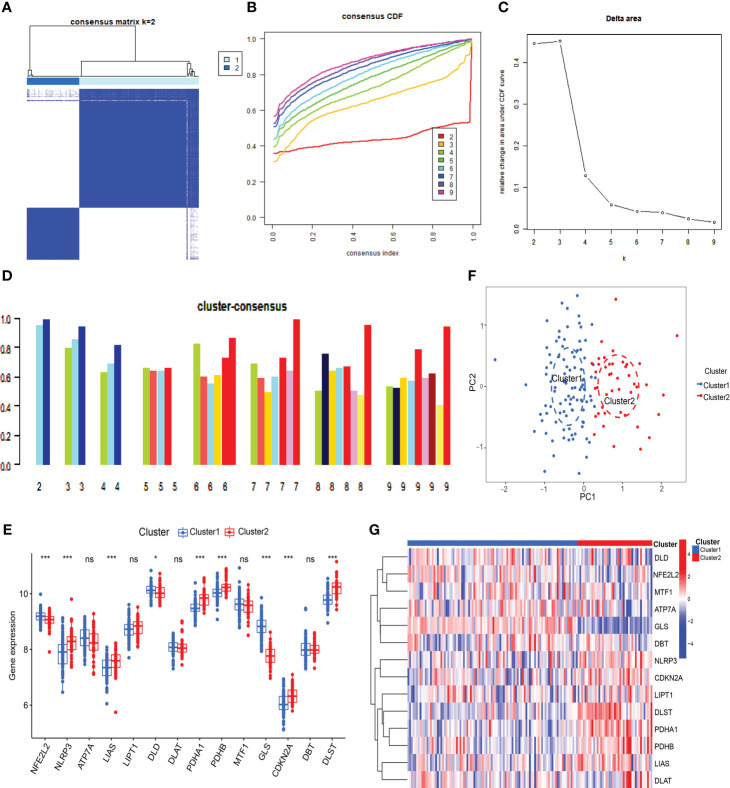
Identification of cuproptosis-related molecular clusters in SLE. **(A)** Consensus clustering matrix when k=2. **(B, C)** Representative cumulative distribution function (CDF) curves **(B)**, CDF delta area curves **(C)**, the score of consensus clustering **(D)**. **(E)** T-SNE visualizes the distribution of two subtypes. **(F)** Boxplots showed the expression of 14 CRGs between two cuproptosis clusters. **(G)** The expression patterns of 14 CRGs were presented in the heatmap. (* *p* ≤ 0.05,*** *p* ≤ 0.001, ns, no significance).

To understand the expression patterns of CRGs in SLE, we conducted a consensus clustering analysis with the expression of the 14 CRGs, and the consensus index fluctuated within a minimal range of 0.2-0.6 (**Figures 3A, B**, **Figure S1**). When k = 2-9, the area under the CDF curve is presented as the difference between two CDF curves (k and k-1) (**Figure 3C**). Furthermore, only when k = 2, the consistency score of all subtypes was> 0.9 (**Figure 3D**). Combining the consensus matrix heatmap, we divided 141 patients into two clusters, including Cluster 1 (n = 98) and Cluster 2 (n = 43) (**Figure 3E**). Patients were clustered by t-distributed stochastic neighbor embedding (t-SNE), showing a significant difference between the two clusters (**Figure 3F**). We generally evaluated the expression difference in 14 CRGs between Clusters 1 and 2 to investigate the molecular characteristics of clusters. Distinct CRG expression patterns were observed between two Clusters. Cluster 1 showed high expression of FDX1, DLD, DLAT, PDHA1, PDHB, and GLS, while Cluster 2 showed enhanced expression of LIPT1, MTF1, CDKN2A, and SLC31A1 (**Figure 3G**). Next, our analysis focused on the immune cell infiltration differences between the two groups, revealing distinct levels of four immune cells. T cells CD8 and M0 macrophages were found to be higher in cluster 2, while neutrophils and M2 macrophages were lower (**Figures S2, 3**). Further analysis using GSVA revealed functional differences in cluster-specific DEGs between the two clusters. Cluster 1 showed up-regulation in Glycine serine and threonine metabolism, Cysteine and methionine metabolism, and Pathogenic Escherichia coli infection signal activity, whereas cluster 2 showed enhancement in inflammation, metabolism, immune response, and TGF-β signal activity (**Figure S4**). Additionally, the functional enrichment results revealed that cluster 1 was significantly associated with positive regulation of ATPase complex of proton transport, mitosis, vitamin D metabolism, and smooth muscle cell apoptosis. In contrast, H3K9me2 demethylase activity, aminophospholipid transferase activity, regulation of RNA binding, and negative regulation of cytoplasmic translation were enriched in cluster 2 (**Figure S5**).

### 3.3 Weighted co-expression network construction and core module selection

**Figure 4:**
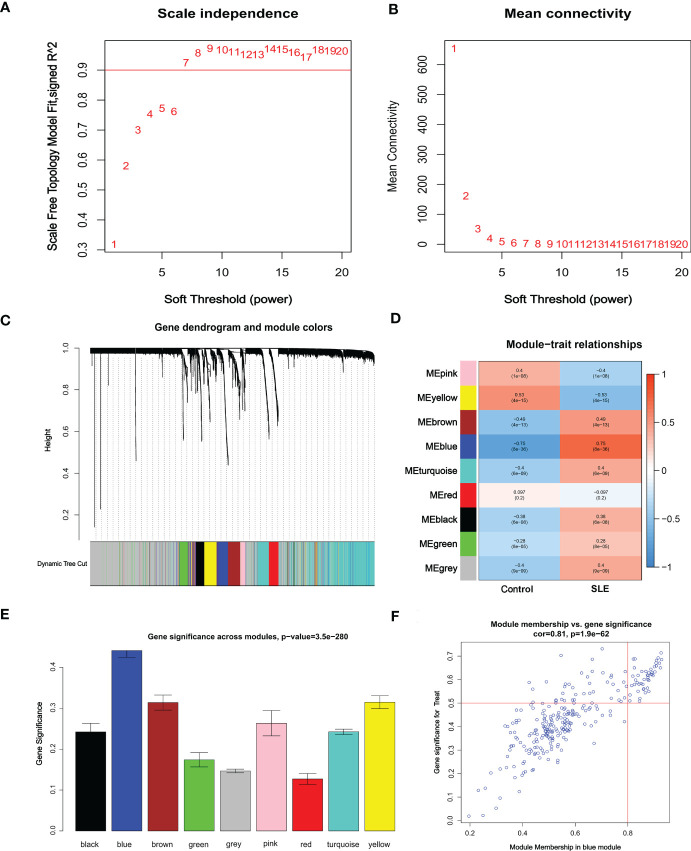
Identification of weighted gene co-expression network modules associated with SLE in GSEM. **(A, B)** Soft threshold selection. **(C)** Dynamic shearing tree merging similar module genes. **(D)** Correlation analysis between module eigengenes and clinical status. **(E)** The correlation between genes and traits between modules. **(F)** The correlation between module membership and genetic importance. cor represents the correlation between GS and MM.

Co-expression network and module were constructed for the Control and SLE groups using the WGCNA algorithm to identify SLE-related core gene modules. We calculated the variance of gene expression in the GSEM dataset and selected the top 25% of genes with the highest variance for further analysis. When Soft was set to 7, scale-free R^2 = ^0.9, and co-expression modules were identified (**Figure 4A**). Altogether 9 co-expression modules with different colors were obtained using the dynamic cut-tree algorithm, and TOM Heatmap was generated (**Figures 4B–D**). Subsequently, genes were consecutively applied in 9 color modules with module-clinical characteristics (Control and SLE) co-expression similarity and adjacency analyzed. The blue module presented the strongest correlation with SLE, including 263 genes (**Figure 4E**, **Table S1**). We also observed the correlation between modules and module-related genes (**Figure 4F**, **Table S2**).

### 3.4 Machine learning model construction and evaluation

**Figure 5:**
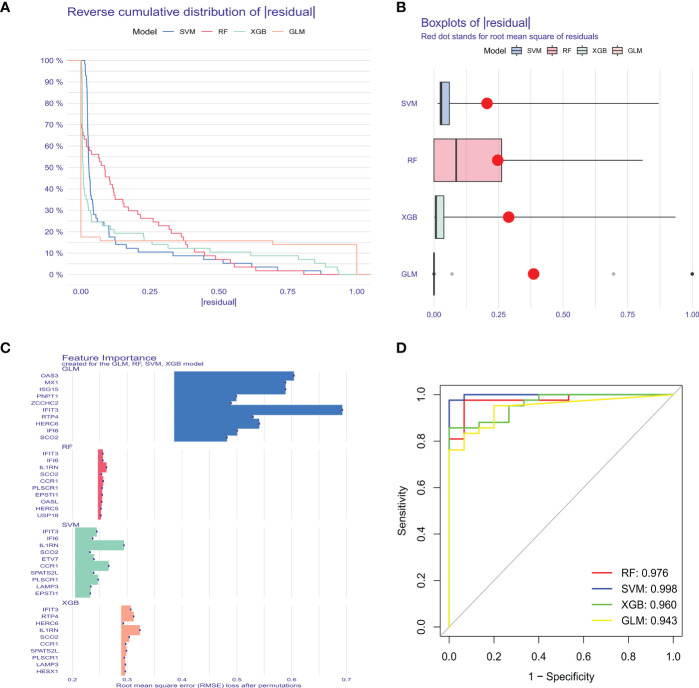
Construction and evaluation of SVM, RF, GLM, and XGB machine models. **(A)** Cumulative residual distribution of each machine learning model. **(B)** Boxplots showed the residuals of each machine learning model. Red dot represented the root mean square of residuals (RMSE). **(C)** The important features in SVM, RF, GLM, and XGB machine models. **(D)** ROC analysis of four machine learning models based on 5-fold cross-validation in the testing cohort.

To further identify critical markers with high diagnostic value, we established 4 machine-learning models based on the blue core module expression profile, including SVM, RF, GLM, and XGB models. The 4 models were explained using the “DALEX” package, and residuals from each model in the training cohort were plotted. The SVM model had a relatively low residual (**Figures 5A, B**). The top 10 important variables of each model were obtained according to root-mean-square error (RMSE) (**Figure 5C**). Moreover, ROC curves of the 5-fold cross-validation were plotted to appraise the diagnostic performance of the 4 machine learning algorithms in the training cohort. SVM model had the highest AUC (SVM, AUC = 0.998; RF, AUC = 0.976; XGB, AUC = 0.960; GLM, AUC = 0.943, **Figure 5D**). Combining those results, the SVM model had the best performance in distinguishing patients from different clusters. The top 5 variables (IFIT3, PLSCR1, CCR1, IL1RN, and ETV7) were selected from the SVM model as critical predictive markers for the following analysis.

**Figure 6:**
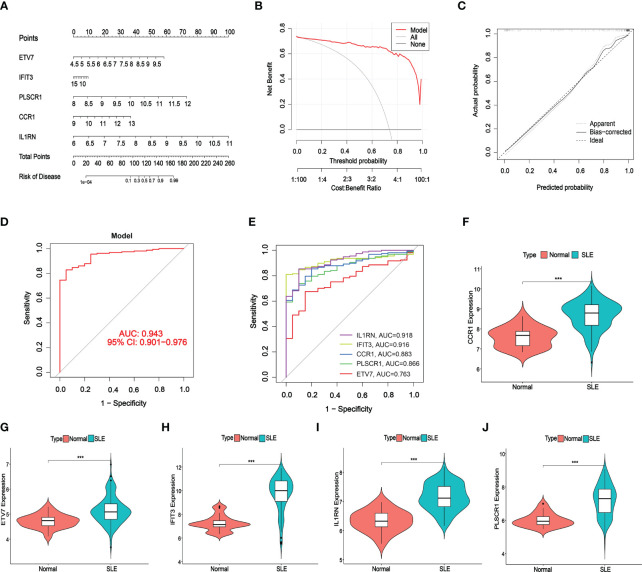
Validation of the 5-gene-based SVW model. **(A)** Construction of a nomogram for predicting the risk of SLE clusters based on the 5-gene-based SVW model. **(B, C)** Construction of calibration curve **(B)** and DCA **(C)** for assessing the predictive efficiency of the nomogram model. **(D, E)** ROC analysis of the 5-gene-based SVW model based on 5-fold cross-validation in GSE72326. **(F-J)** The expression levels of 5-genes were verified with validated dataset GSE72326. (*** *p* ≤ 0.001).

A nomogram was constructed to assess the predictive efficiency of the SVM model using 141 SLE cases to predict the risk of cuproptosis aggregation (**Figure 6A**). The predictive efficiency of the nomogram was assessed using the calibration curve and DCA. According to the calibration curve, in the SLE cluster, the error between the actual risk and the predicted risk was very small (**Figure 6B**). DCA revealed that the nomogram had high accuracy that can provide evidence for clinical decisions (**Figure 6C**). Subsequently, we validated the predictive capability of the 5 core markers using the validation cohort GSE72326. The ROC curve revealed that the predictive model with 5 core markers had a favorable performance with an AUC of 0.943 (**Figure 6D**), suggesting that our diagnostic model can effectively discriminate SLE patients from normal cases. Meanwhile, the diagnostic value of single markers was verified as well, and IL1RN had the highest AUC (AUC = 0.918, **Figure 6E**). The expression of the 5 core diagnostic markers was all upregulated in SLE patients (**Figures 6F–J**).

### 3.5 CeRNA network establishment of core diagnostic markers

**Figure 7:**
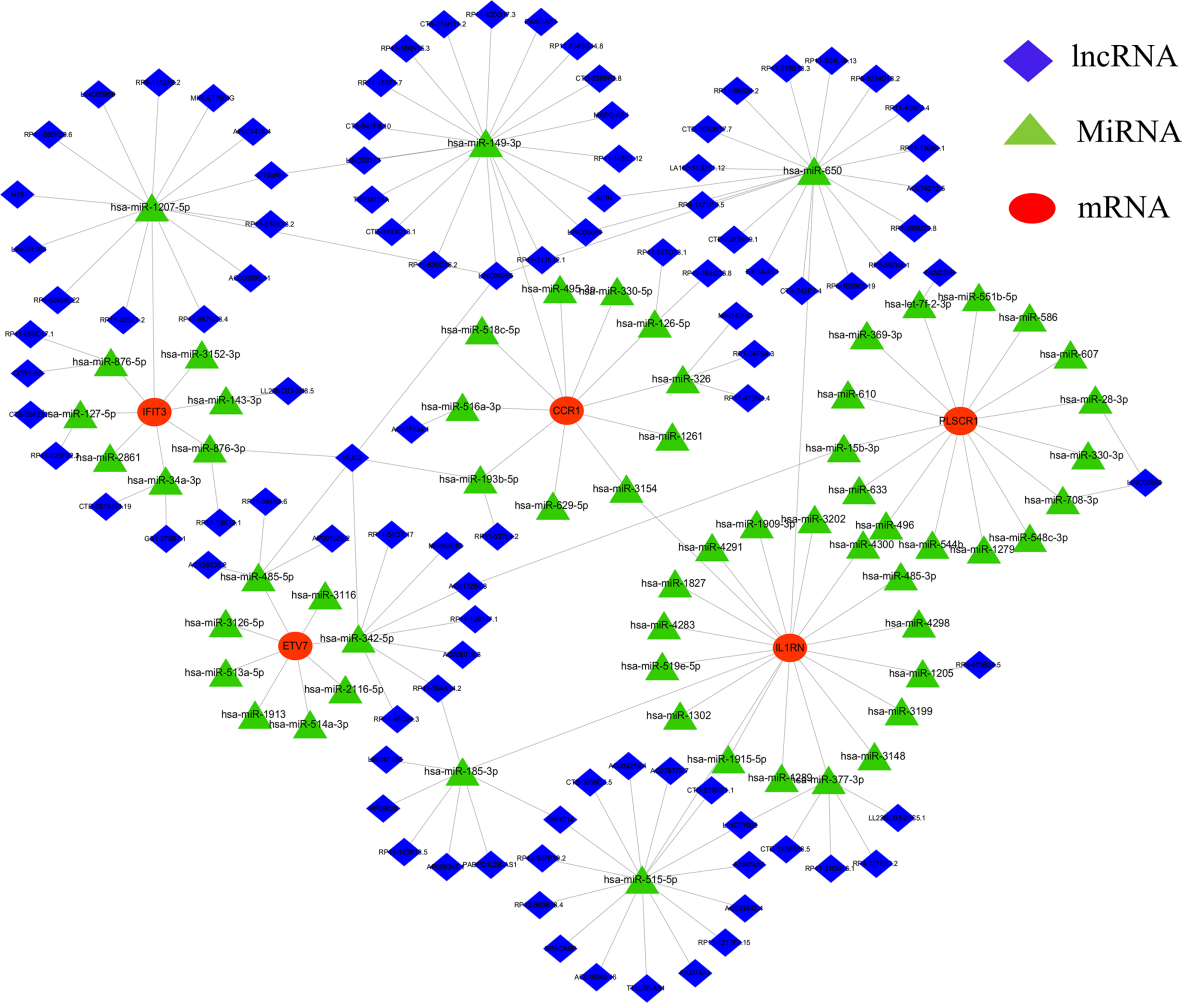
lncRNA-miRNA-mRNA regulatory network. The red represent the mRNAs, the green represent the miRNAs and the blue represent the lncRNAs.

A CeRNA network was constructed using miRanda, targetScan, miRDB, and SpongeScan databases with 5 core diagnostic markers. The CeRNA network contains 166 nodes (5 core diagnostic markers, 61 miRNAs, and 100 lncRNAs) and 175 lines (**Figure 7**). Eventually, 47 lncRNAs can competitively bind with IL1RN regulated by hsa-miR-650, hsa-miR-515-5p, hsa-miR-377-3p, hsa-miR-185-3p, and hsa-miR-1205, among which lncRNA SNHG14 can simultaneously target hsa-miR-515-5p and hsa-miR-185-3p. 23 lncRNA can target IFIT3 regulated by hsa-miR-876-3p, hsa-miR-127-5p, hsa-miR-34a-3p, hsa-miR-143-3p, hsa-miR-1207-5p, and hsa-miR-876-5p. Additionally, 19 lncRNA can regulate CCR1 expression by competitively binding with hsa-miR-149-3p. In the ceRNA network of PLSCR1, LINC00662 can bind with hsa-miR-28-3p and hsa-miR-708-3p to regulate PLSCR1. 8 lncRNAs competing with hsa-miR-342-5p to regulate ETV7 expression.

### 3.6 Prediction of targeted drugs for core diagnostic markers

**Figure 8:**
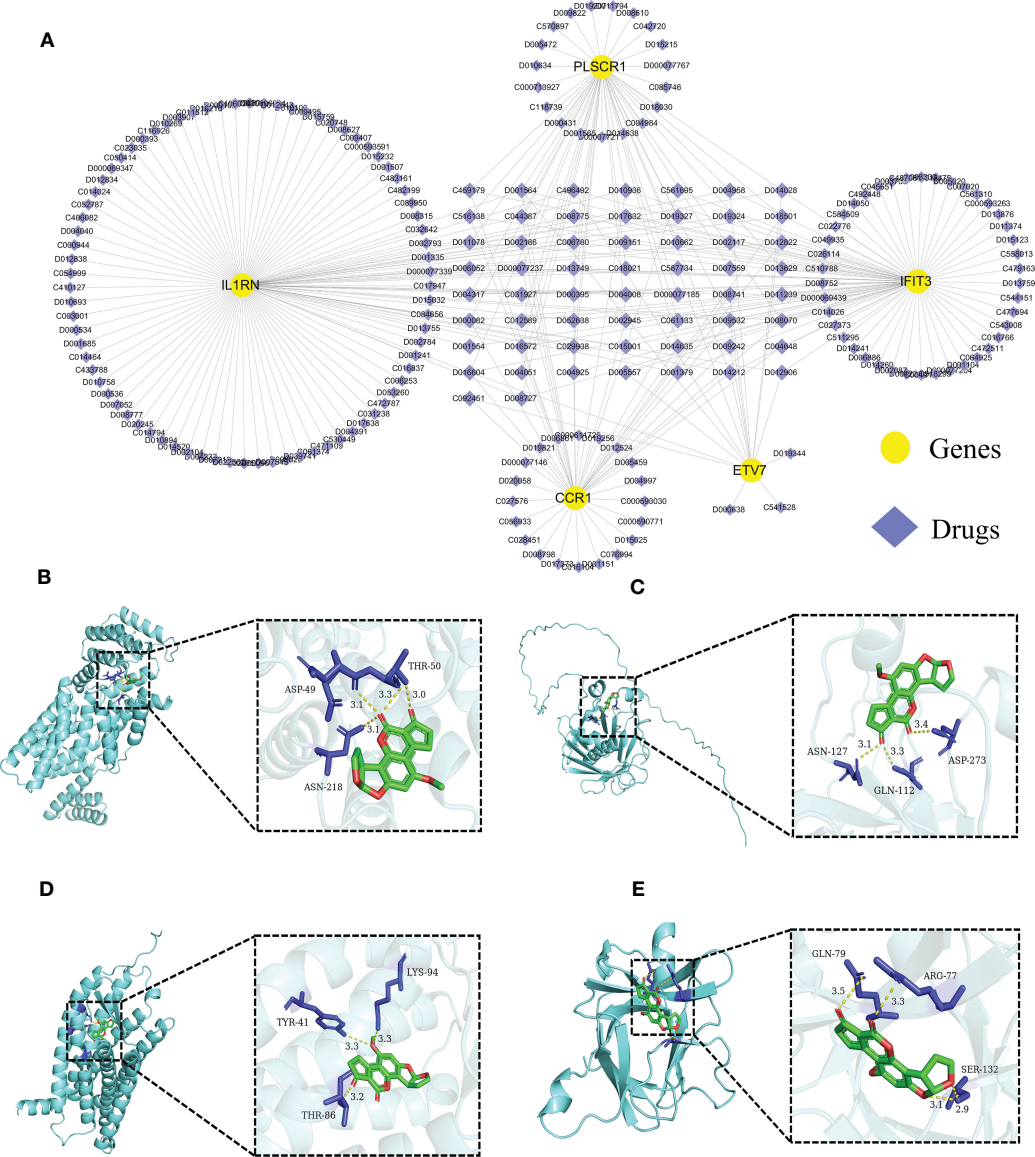
Predicting the drugs targeting the diagnostic biomarkers. **(A)** Drug-gene network. **(B- E)** Molecular docking analysis. Aflatoxin B1 was docked with IFIT3, PLSCR1, CCR1, and IL1RN.

We further predicted drugs of the 5 core diagnostic markers using the CTD database, extracted drug-marker interactions, and constructed a drug-marker network containing 226 knots and 319 edges, in which 5 core diagnostic markers and 221 drugs were included. The results were visualized using the Cytoscape software (**Figure 8A**, **Table S3**). Drug detection showed that D00156 (Benzo (a) pyrene), D016604 (Aflatoxin B1), D014212 (Tretinoin), and D009532 (Nickel) could simultaneously act on the 5 core diagnostic markers. And a molecular docking was performed between drugs and predicted molecular targets (**Figures 8B–E**, **Figure S7**, **Table S4**).

## 4 Discussion

At present, the pathogenesis of SLE has not been fully elucidated. It is widely accepted that SLE develops on a specific genetic background and epigenetic modifications upset the immune system balance, leading to aberrant immune cell proliferation, massive production of autoantibodies, and eventually multiple organ damage (32, 33). However, a single causal gene has not been identified. Conversely, currently, mounting studies reported that multigenic interactions were closely related to SLE initiation and multi-organ involvement (34, 35). Therefore, searching for core molecular clusters is crucial to instruct SLE diagnosis and individualized treatment. Cuproptosis is a newly reported copper-dependent cell death form, mainly manifesting mitochondrial aggrecanase lipoylation, and is closely associated with disease progression (36). However, the specific mechanism and regulatory role of cuproptosis in various diseases have not been further delved into. Hence, we endeavored to elucidate the role of CRGs in SLE phenotype and immune microenvironment.In this study, we analyzed the expression profile of CRGs in the peripheral whole blood of SLE patients. The aberrant gene expression level in SLE patients was higher than in normal individuals, suggesting that CRGs play a significant role in SLE initiation. The correlation among CRGs was calculated to reveal the relationship between CRGs and SLE. Significant synergistic or antagonistic effects were identified among CRGs. Meanwhile, differentiation was observed in immune cell abundance between the control group and SLE patients. SLE patients exhibited a higher infiltration level of neutrophils, memory B cells, plasma cells, and activated DC. Neutrophils play a pathogenic role in multiple AIDs, including SLE (37). Neutrophils can induce plasmacytoid dendritic cells (pDC) to generate interferon (IFN), thereby advancing disease progression (38). Furthermore, the complex genetic background of SLE patients could provide multiple amplification steps for the perpetuation and subsequent pathogenicity of neutrophil-pDC interactions (39). ISG15 in neutrophils may also induce the production of Th1 lymphocytes with pro-inflammatory properties (40). Moreover, by applying unsupervised clustering analysis, we confirmed two distinct clusters based on CRG expression to illustrate the different regulatory patterns of SLE patients. These results demonstrated that CRGs might be the key factors that regulate SLE occurrence and immune infiltration status.

Machine learning is a multidisciplinary discipline, and modeling using the machine learning method can explore the underlying value of data. Additionally, machine learning plays an indispensable role in effectively utilizing data and supporting clinical decisions. In this study, we compared the predictive performance of the 4 machine learning methods (SVM, RF, GLM, and XGB), and constructed a predictive model based on SVM (best performance, AUC = 0.998), suggesting that the SVM model had favorable performance when predicting SLE. An SVM model was established using the 5 important factors (IFIT3, PLSCR1, CCR1, IL1RN, and ETV7). Research showed that IFIT3 belongs to the interferon-induced protein family and is an anti-viral protein (41). IFIT3 can block the synthesis of type I IFN and other inflammatory cytokines via the cGAS/STING pathway (42). PLSCR1 shows increased expression in multiple systemic AIDs, such as primary antiphospholipid syndrome, rheumatoid arthritis, idiopathic inflammatory myopathies, and SLE (43, 44). A correlation was identified between PLSCR1 expression and type I interferon-stimulated genes (45), and PLSCR1 is highly expressed in neutrophils, DC, and macrophages (46). CCR1 is a member of the β-chemokine receptor family and can interact with numerous ligands, such as CCL5, and suppressing CCR1 could improve lupus nephritis progression in New Zealand black/white mice (47). IL1RN is a natural IL-1 inhibitor that can regulate multiple IL-1-related immune and inflammatory responses. IL1RN polymorphism is a factor that affects SLE severity, and IL1RN might be a potential biomarker for SLE (48). Transcription factor ETV7 exhibited elevated expression in SLE (49), which might be induced by IFN-α/γ (50, 51). The SVM model accurately predicted SLE in the validation cohort (AUC = 0.943), providing new insights for SLE diagnosis. More importantly, a nomogram was plotted based on IFIT3, PLSCR1, CCR1, IL1RN, and ETV7 for diagnosing SLE subtypes. The nomogram displayed significant predictive value, suggesting that the model has clinical utility.

In addition, we constructed a CeRNA network using the 5 core diagnostic markers to explore the regulatory mechanism of the core markers. MicroRNA (miRNA) is one of the major epigenetic regulators of SLE-related genes. Remarkable research progress has been made in miRNA-based biomarkers and therapies (52). The CeRNA network illustrated that lncRNA SNHG14 could simultaneously interact with hsa-miR-515-5p and hsa-miR-185-3p, lncRNA SNHG14 could participate in the production of proinflammatory cytokines in rheumatoid arthritis by regulating the MINK1/JNK pathway (53). It was reported that hsa-miR-515-5p regulated WISP1 expression, inhibited the TLR4/JNK signaling pathway, and reduced apoptosis in fibroblast-like synoviocytes (RAFLS) of rheumatoid arthritis (54). The hsa-miR-185-3p modulating transcription factor Foxo1 plays a foremost role in AIDs and can serve as a diagnostic marker of SLE (55–57). We also predicted diagnostic markers-associated drugs using the CTD database, constructed the drug-gene network, and predicted targets of action by constructing molecular docking models. This offered a reference for devising new protocols or investigating potential pathogenic factors for SLE (58). For instance, tretinoin simultaneously targets 5 core diagnostic markers and is a reactive derivative of vitamin A, which can regulate cellular proliferation, differentiation, and maturation (59). Previous studies have indicated that the imbalance of Th17/Treg cells was closely related to the pathogenesis and disease activity of SLE (60). Tretinoin can regulate the balance of Th17/Treg cells by downregulating IL-6Ra expression, which affects the binding of IL-6 to IL-6Ra and gp130, leading to the recruitment of STAT3 and promotion of its phosphorylation to induce ROR γt expression, and ultimately inhibits Th17 cell differentiation and promotes Treg cell proliferation (61, 62). Tretinoin is a potent inhibitor of Pin1, effectively blocking the TLR-7/TLR-9/Pin1/IRAK-1/IRF-7 signaling pathway by inhibiting and degrading activated Pin1, making it an attractive candidate for treating SLE, as Pin1 plays a key role in preventing the progression of the disease (63). Although the potential therapeutic effects of tretinoin are still being explored in this field, it has gained increasing attention, and more research can be carried out to explore its potential therapeutic effects and bring more medical progress.

Nevertheless, there are some limitations to this study. First, this current study is based on bioinformatics, and additional clinical data and experiments are required to verify CRG expression levels. Second, more detailed clinical characteristics are required to identify the performance of the predictive model, and more SLE samples are required to demonstrate the accuracy of the CRG-based model. The correlation between CRGs and immune response needs to be further explored.

## Data availability statement

The original contributions presented in the study are included in the article/**Supplementary Material**. Further inquiries can be directed to the corresponding author.

## Author contributions

WL and YS conceived the project and designed the study. YoW and XG analyzed the data and wrote the manuscript. YL and YuW interpreted the data and provided practical resources. MY revised the paper. All authors contributed to the article and approved the submitted version.
