# Supplementary material for: Cuproptosis-related gene identification and immune infiltration analysis in systemic lupus erythematosus
Source: Front Immunol. 2023 May 29;14:1157196. doi: 10.3389/fimmu.2023.1157196 (PMC10258330; doi:10.3389/fimmu.2023.1157196)
Supplement: Supplementary file 1 [file DataSheet_1.pdf]

Supplementary Material

1. Supplementary Figures

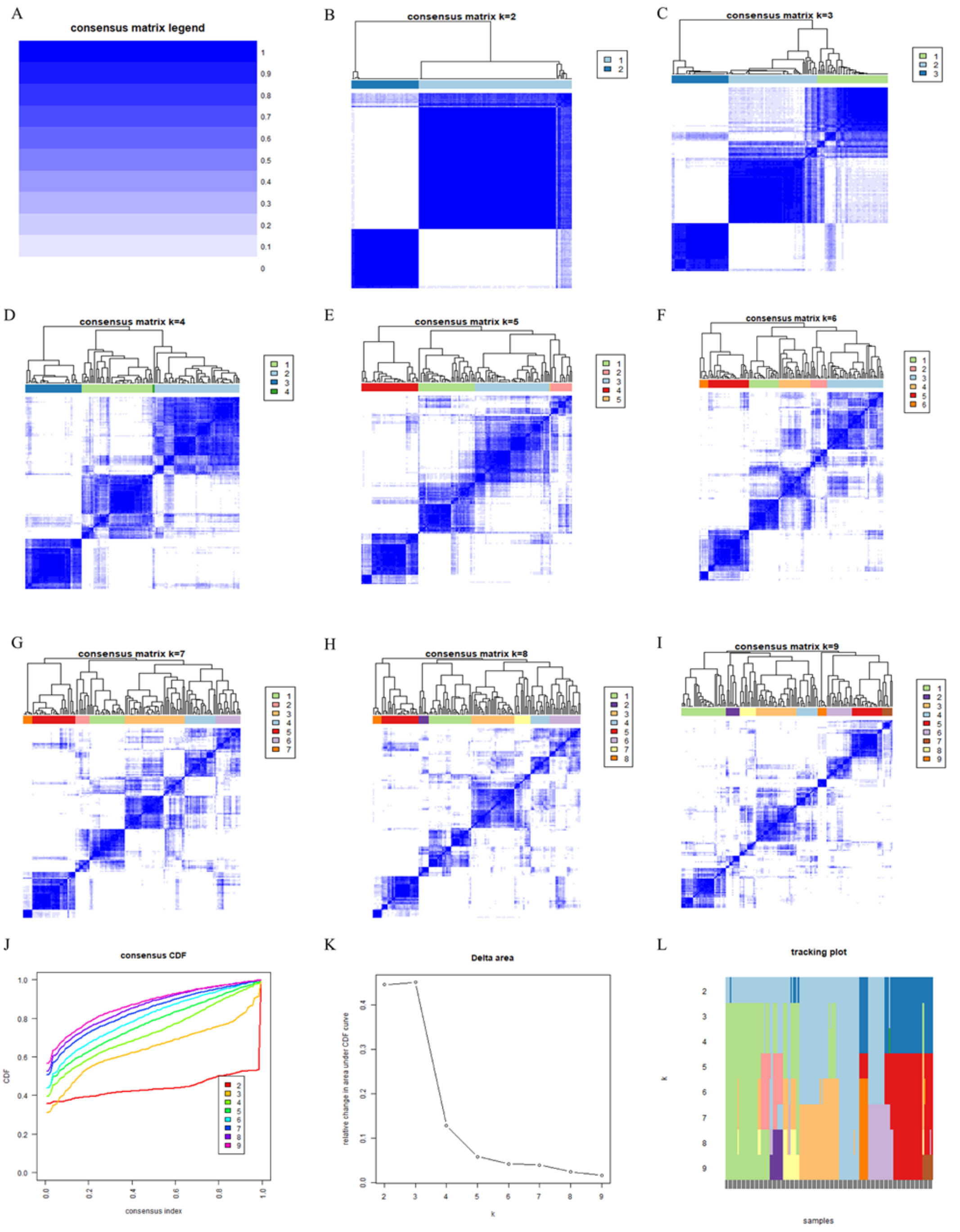

Supplementary Figure 1: Identification of cuproptosis-related molecular clusters in SLE. (A-I) Consensus clustering matrix when k = 2. (J) Uniform clustering cumulative distribution function (CDF) with k from 2 to 9. (K) The change of area under CDF curve with k from 2 to 9. (L) The tracking plot showed the relationship between samples and clusters.

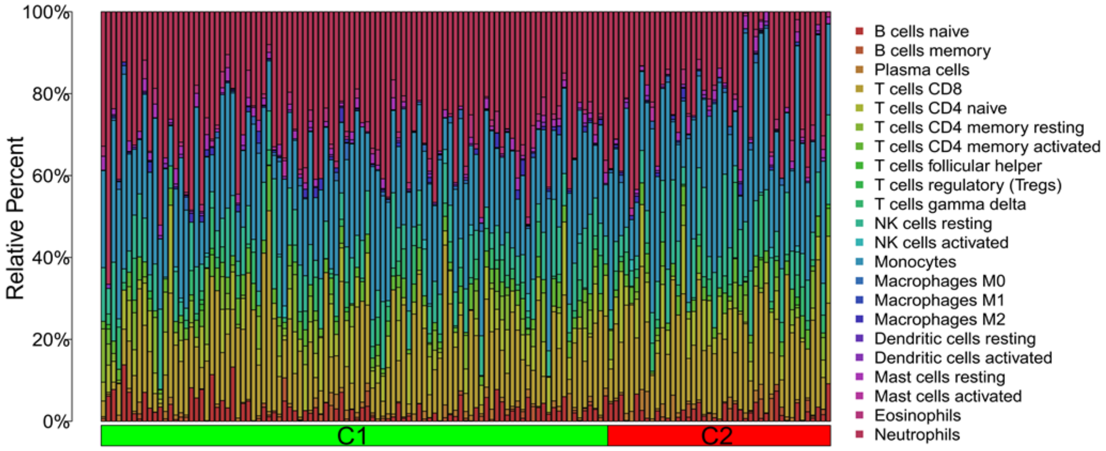

Supplementary Figure 2: The relative abundances of 22 infiltrated immune cells between two cuproptosis clusters.

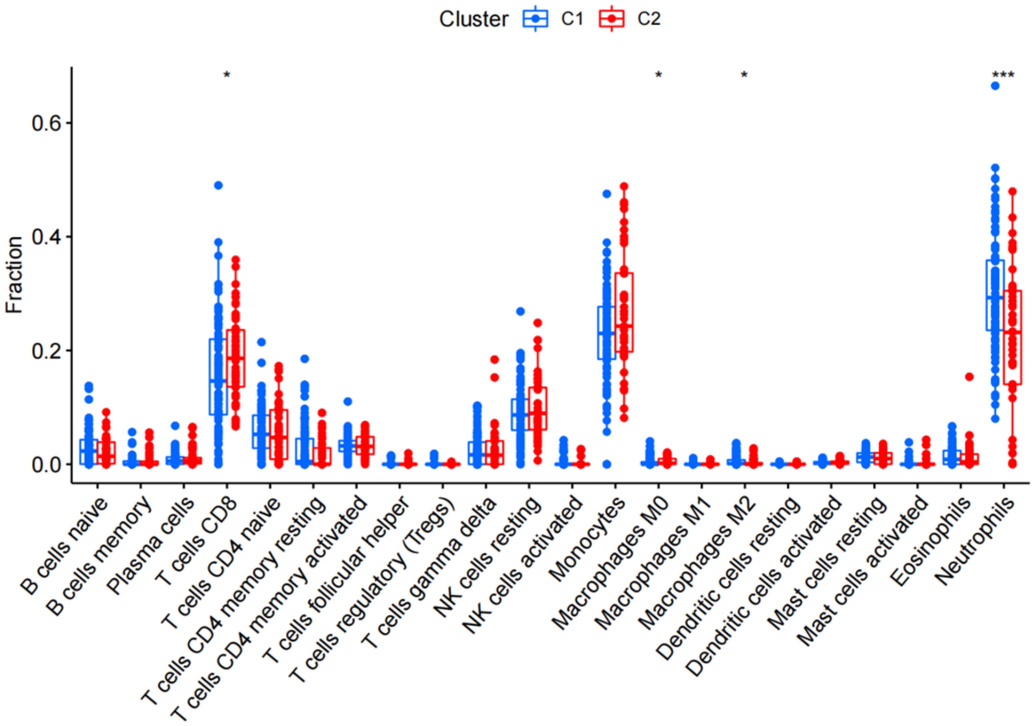

Supplementary Figure 3: Boxplots showed the differences in immune infiltrating between two cuproptosis clusters. $*p < 0.05$, $***p < 0.001$.

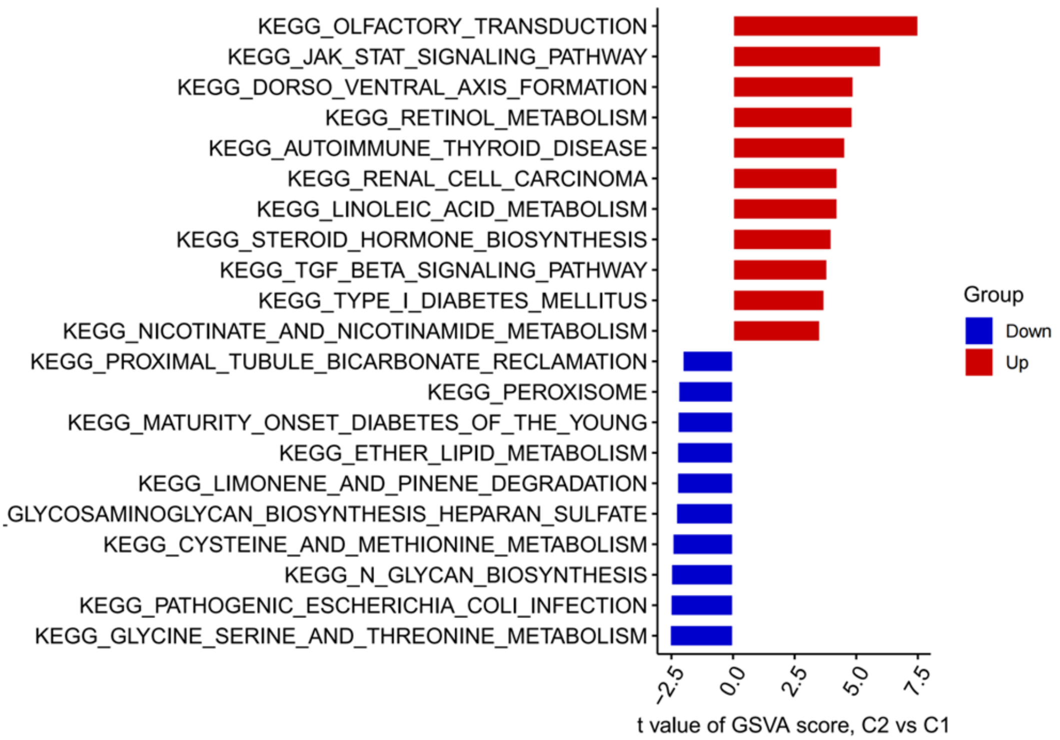

Supplementary Figure 4: Differences in hallmark pathway activities between cluter1 and cluster2 samples ranked by t-value of GSVA method.

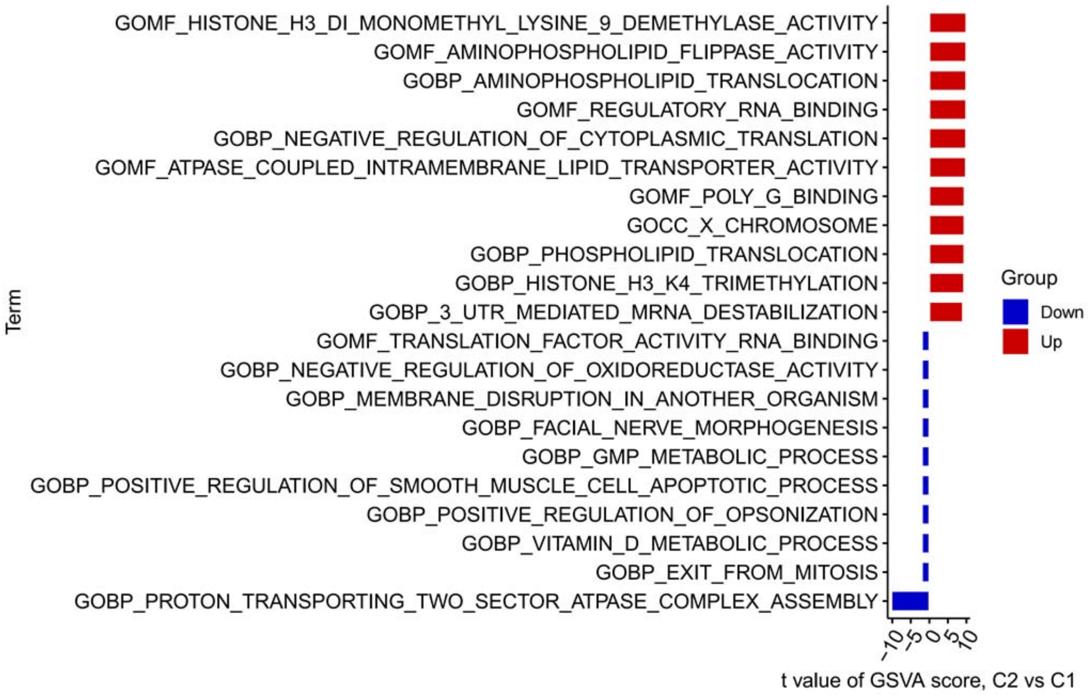

Supplementary Figure 5: Differences in biological functions between cluter1 and cluster2 samples ranked by t-value of GSVA method.

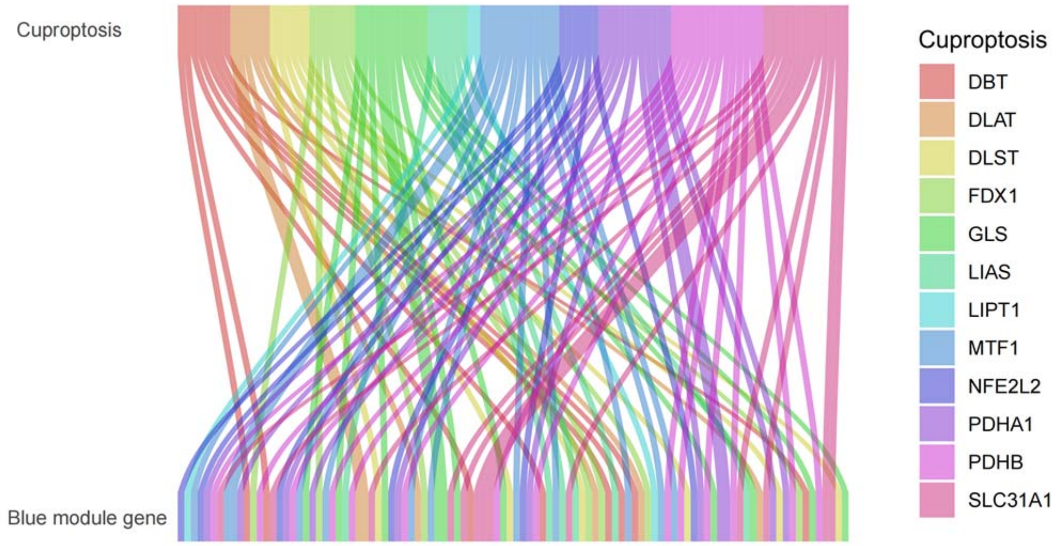

Supplementary Figure 6: The Sankey diagram demonstrates the connection degree between CRGs and blue module genes in SLE.

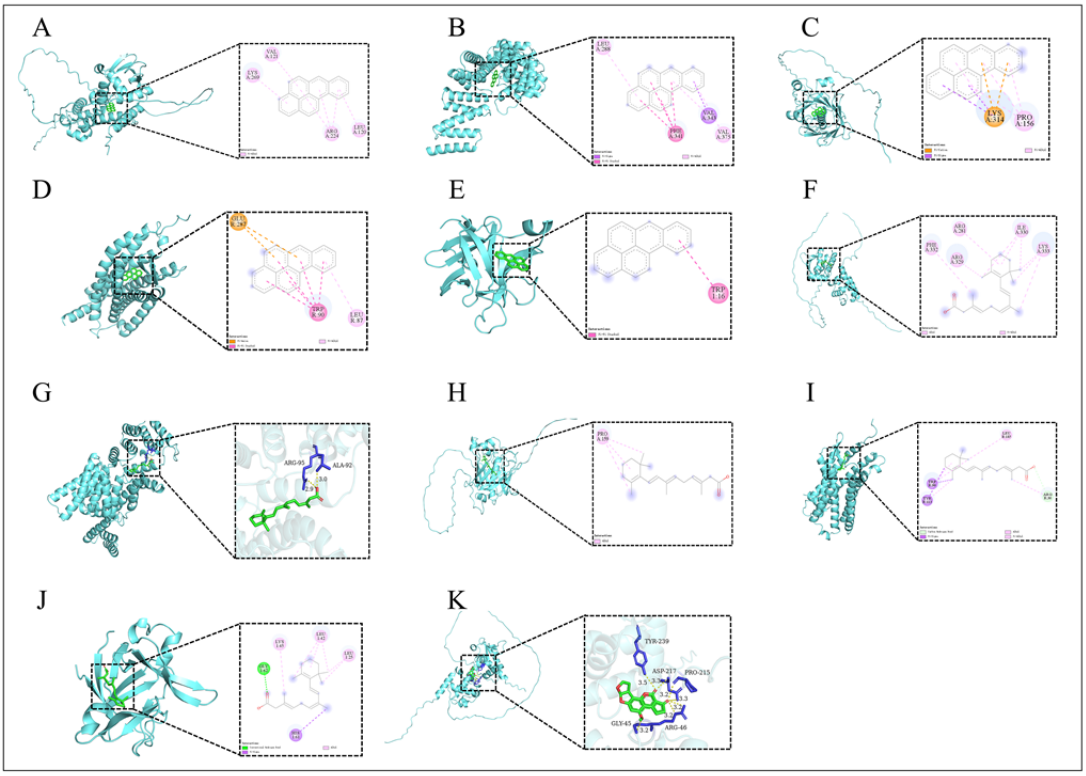

Supplementary Figure 7: Predicting the drugs targeting the diagnostic biomarkers. (A- E) Molecular docking analysis. Benzo(a)pyrene was docked with ETV7, IFIT3, PLSCR1, CCR1, and IL1RN. (F- J) Molecular docking analysis. Tretinoin was docked with ETV7, IFIT3, PLSCR1, CCR1, and IL1RN. (K) Molecular docking analysis. Aflatoxin B1 was docked with ETV7.

2. Supplementary Tables

Supplementary Table 1. Blue module genes
Supplementary Table 2. Blue module core genes
Supplementary Table 3. Drug sensitivity
Supplementary Table 4. Molecular docking of binding energy
